# Paracentral Acute Middle Maculopathy Associated With Migraine With Aura in Pregnancy

**DOI:** 10.7759/cureus.75072

**Published:** 2024-12-03

**Authors:** Chathuranka De Silva, Joanna Da Costa

**Affiliations:** 1 Newcastle Eye Centre, Royal Victoria Infirmary, Newcastle, GBR

**Keywords:** migraine aura, oct angiography, paracentral acute middle maculopathy, pregnancy, retinal diseases

## Abstract

Paracentral acute middle maculopathy (PAMM) is a form of ischaemic maculopathy involving infarction of the inner nuclear layer secondary to deep retinal capillary plexus ischaemia. Risk factors include retinal vascular disease and systemic vascular risk factors, which can predispose the eye to ischaemia. Recent reports now show associations with pregnancy and also migraine episodes. We report an interesting case of a young patient developing PAMM following a migraine with an aura episode during an uncomplicated pregnancy.

## Introduction

Paracentral acute middle maculopathy (PAMM) was first described by Sarraf et al. in 2013 in a small case series of patients with acute scotomas, parafoveal white-grey lesions, and a band-like lesion in the inner nuclear layer (INL) and outer plexiform layer (OPL) on spectral domain optical coherence tomography (SD-OCT) [1]. Originally, authors described PAMM as a variant of acute macular neuroretinopathy (AMN), which conversely affects the outer retinal layers [1].

The aetiology of PAMM is still not fully understood but is thought to be related to reduced blow flow in the deep capillary plexus. PAMM can be idiopathic or secondary to underlying retinal vascular diseases and systemic vascular diseases as well as vasopressor exposure including caffeine intake and the use of sympathomimetics. More recent reports have found that pregnancy alone can be a risk factor as well as migraine episodes. The exact pathogenesis of PAMM is still not clearly defined.

We report a young patient developing PAMM following a migraine with an aura episode during an uncomplicated pregnancy and explore what the current literature suggests may be the underlying mechanisms for this event.

## Case presentation

A 23-year-old with no co-morbidities presented during the 18th week of her first pregnancy with a right eye scotoma. Preceding this, she had an episode of positive visual aura in both eyes lasting around 30 minutes followed by a unilateral headache lasting a few hours. She reported no other neurological deficits. She had no significant past medical history and was on no regular medications. She had no prior history of migraines. Her caffeine intake was above average (around 500-600 micrograms/day) but she denied the use of any sympathomimetic drugs. 

**Figure 1:**
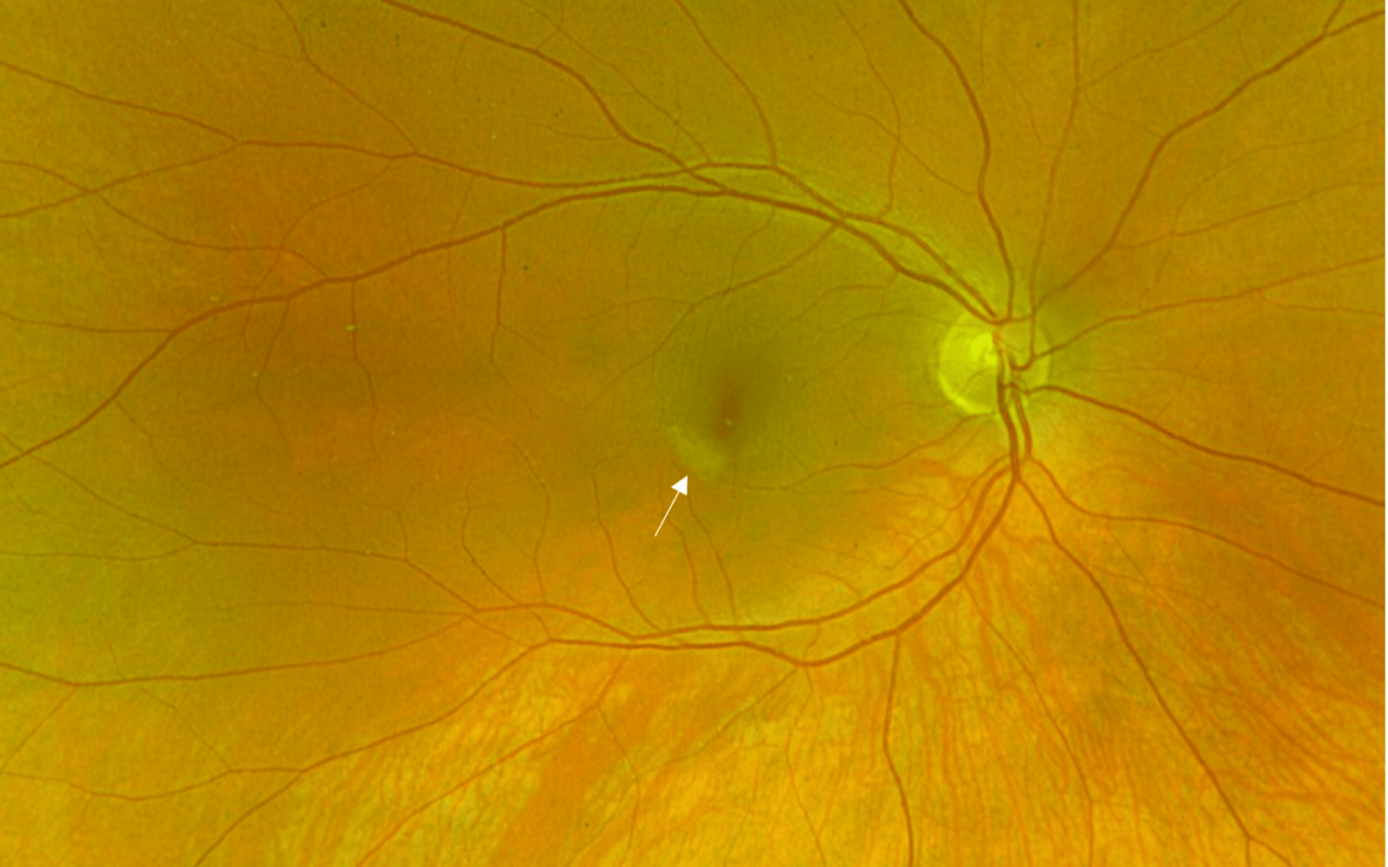
Optos image of the right eye fundus showing parafoveal wedge-shaped grey lesion (arrowed)

**Figure 2:**
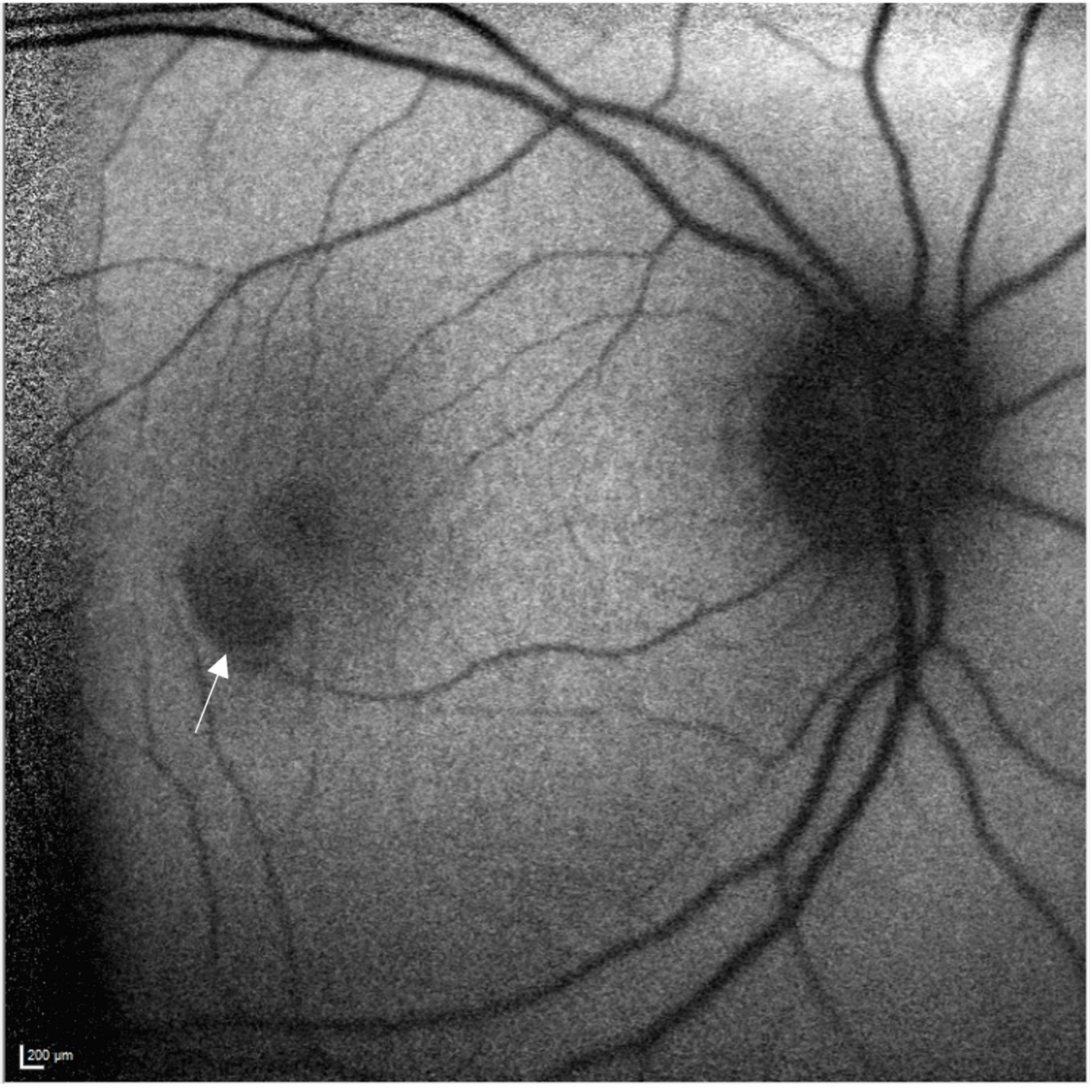
Heidelberg fundus autofluorescence of the right eye (arrowed)

**Figure 3:**
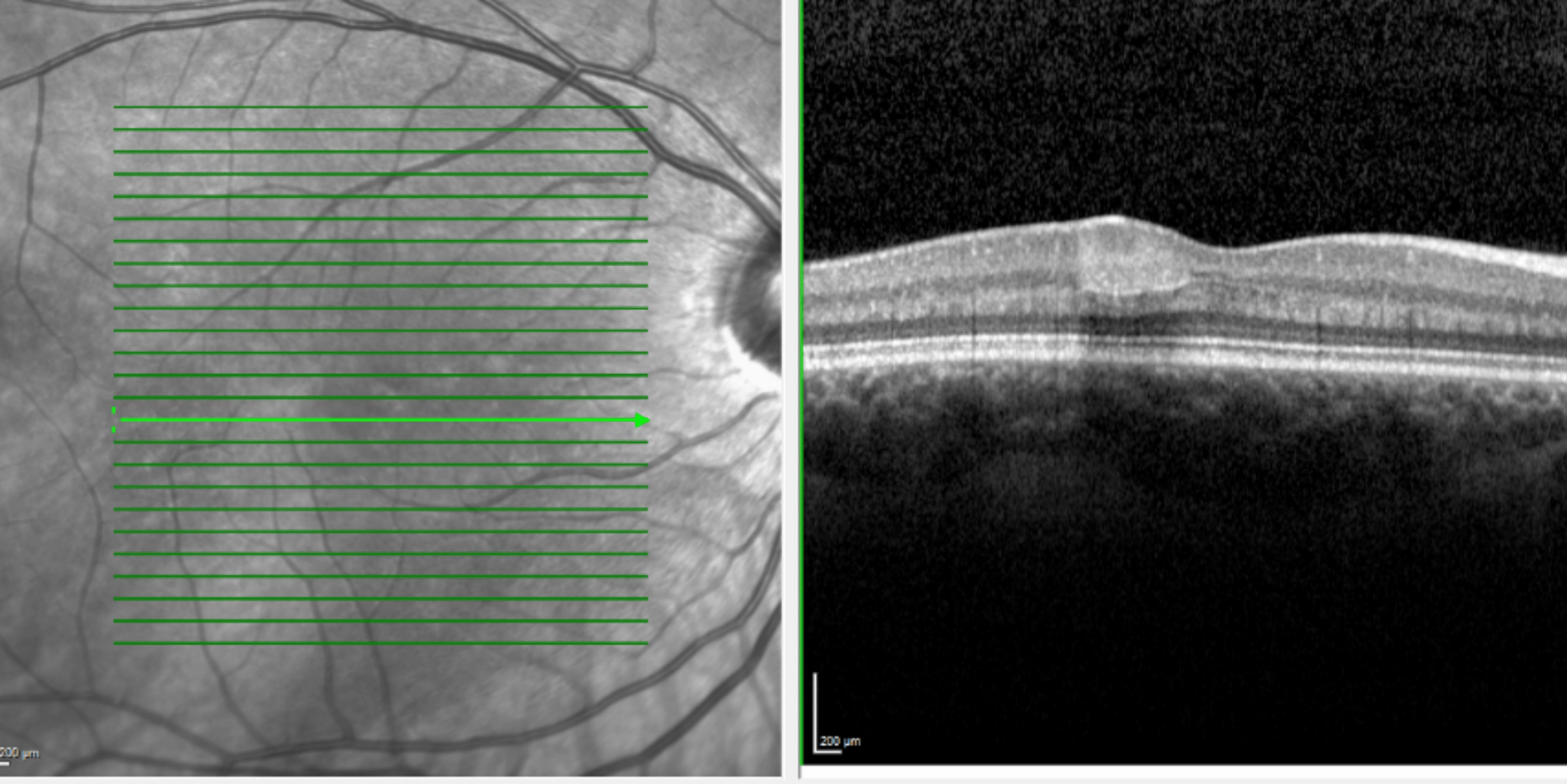
Heidelberg SD-OCT of the right eye macula at presentation SD-OCT, spectral domain optical coherence tomography.

On examination, visual acuity was 6/6 in both eyes and she had healthy anterior segments. Intraocular pressures were 14 mmHg in both eyes. On fundoscopy, she was noted to have a pale grey wedge-shaped lesion temporal to the fovea in the right eye (Figure 1). Fundus autofluorescence showed corresponding hypoautofluorescence of the lesion (Figure 2). SD-OCT showed a correlating hyper-reflective band-like lesion encompassing the INL and OPL (Figure 3). The rest of the fundoscopy was normal in both eyes. Based on her SD-OCT finding, a diagnosis of PAMM was made.

Her blood pressure was recorded as 132/88 mmHg and her blood sugar was 5.2 mmol/L. Full blood count, erythrocyte sedimentation rate, c-reactive protein, coagulation, and lipid profile were unremarkable. On review with her maternity team, she was found to have a strong family history of deep vein thrombosis during pregnancy and was started on prophylactic tinzaparin at 28 weeks for the remainder of her pregnancy.

**Figure 4:**
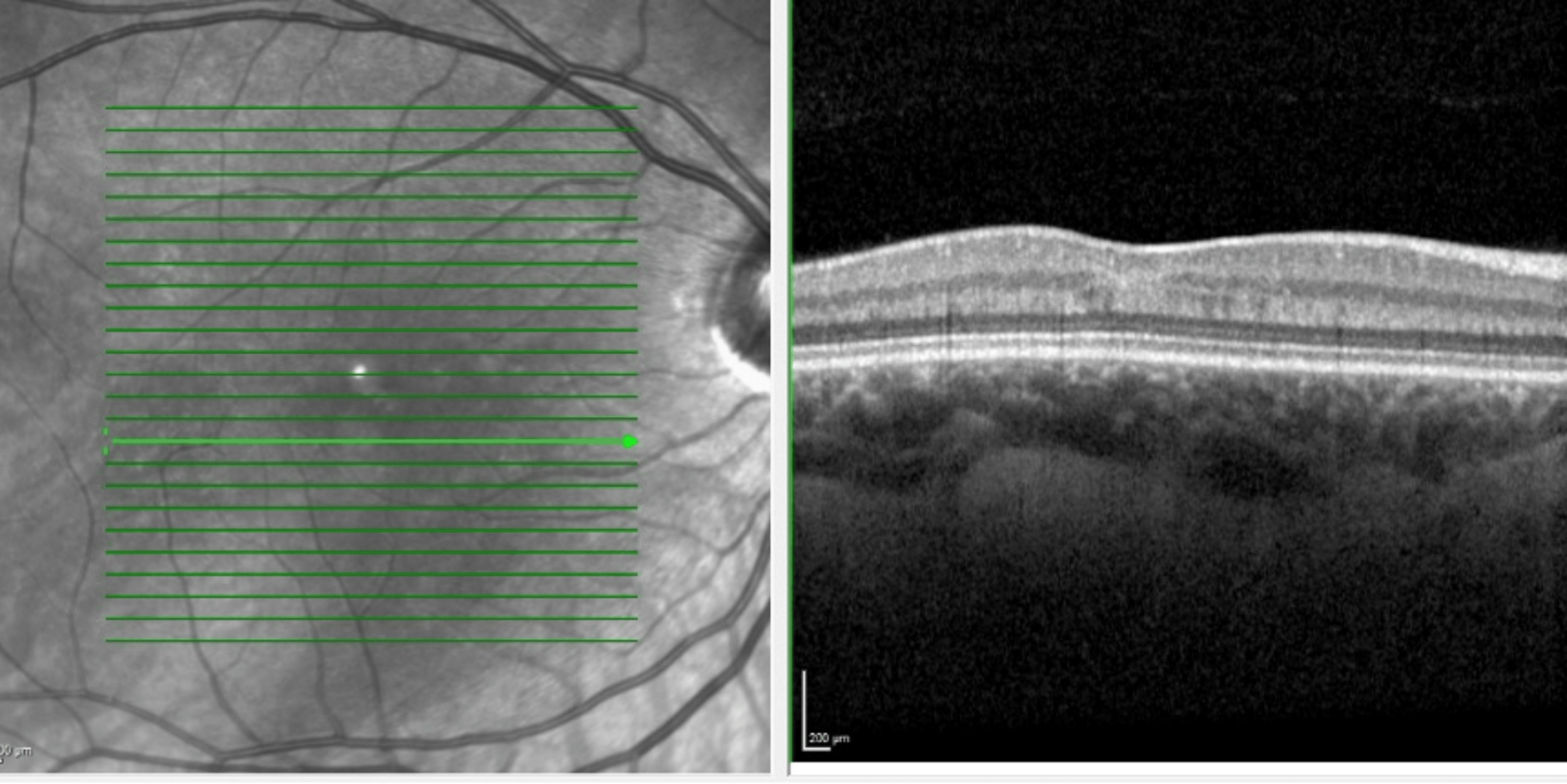
Heidelberg SD-OCT of the right eye macula 11 weeks after presentation SD-OCT, spectral domain optical coherence tomography.

**Figure 5:**
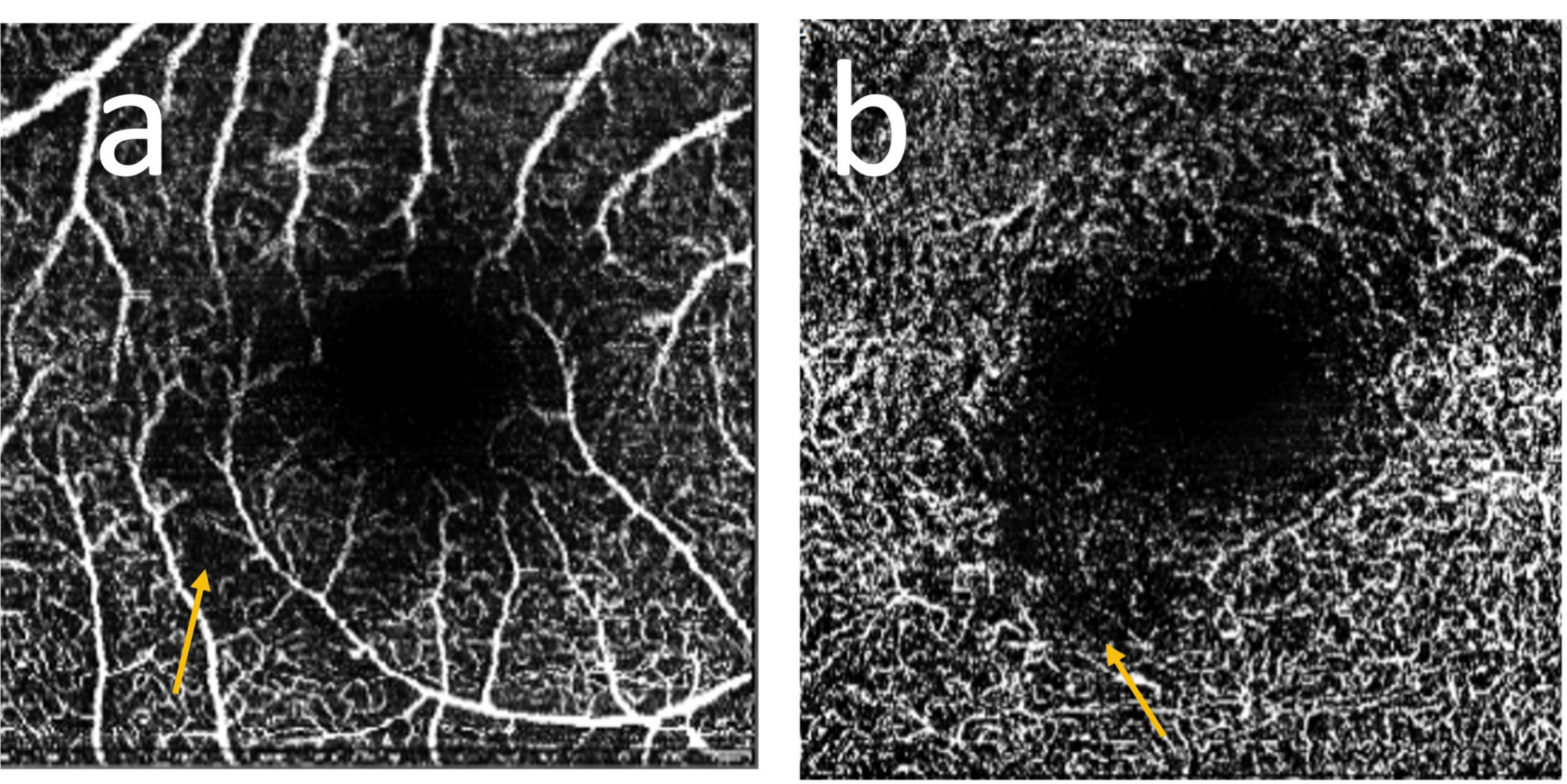
Heidelberg en-face OCT-angiography of the right eye: (a) superficial plexus; (b) deep plexus; arrows depict areas of capillary loss OCT, optical coherence tomography.

She was seen by ophthalmology during the 29th week of her pregnancy. The scotoma had shrunk in size considerably and she was largely asymptomatic. She reported no further migraine episodes. Fundoscopy was now normal. SD-OCT imaging showed a resolved hyperreflective band but thinning of the INL and irregularity of the OPL were noted at that time (Figure 4). OCT-angiography (OCT-A) showed in the right eye subtle areas of capillary loss in the superficial plexus and a larger more obvious patch of capillary deficit in the deep plexus (Figure 5). 

The patient went on to have an uneventful birth at 39+2 weeks. She continued to have a small scotoma but barely noticeable post-partum. Her subsequent SD-OCT and OCT-A continued to show stable appearances. Her patch of deep capillary plexus dropout has not improved thus far. She has not suffered any further migraine episodes.

## Discussion

PAMM typically is an ocular condition presenting with sudden-onset single or multiple scotomas. On examination, one would see wedge-shaped grey retinal lesions and a correlating SD-OCT hyperreflective band in the INL and OPL [1]. OCT-A in the acute phase can show either preservation or loss of vascular flow [2]. If there is evidence of vascular loss, this is usually in the intermediate or deep capillary plexus; however, there have also been reports where acutely the superficial plexus can have capillary loss or distortion of the foveal avascular zone contour [3]. 

Currently, there is no treatment advised for PAMM. The lesions regress over time and symptoms usually improve with partial resolution, although in rare cases the scotoma can persist and be visually debilitating. On SD-OCT, the hyperreflective band resolves within months, with thinning and irregularity observed in the INL and OPL [1,4]. An excavated change in the inner retinal surface, accompanied by thickening of the outer nuclear layer, may also be observed [5]. On OCT-A, one would see vascular loss with capillary pruning or dropout predominately in the deep capillary plexus, sometimes with dilatation of the capillaries surrounding these areas [6]. 

The primary management of PAMM involves a thorough investigation of potential risk factors. While PAMM can be idiopathic, it is often linked to systemic conditions such as cardiac disease, carotid stenosis, diabetes, hypertension, vasculitis (including giant cell arteritis), and retinal vascular disorders like central and branch retinal vein occlusions, sickle cell disease, and Purtscher's retinopathy [5,7]. Reported exogenous risk factors include medications (such as amphetamines, caffeine, vasopressors, and oral contraceptives), hypovolemia, orbital compression injury, vaccinations, viral prodromes, and upper respiratory infections (such as H1N1 and SARS-CoV-2) [8,9].

Recent reports have shown associations with pregnancy alone. To our knowledge, this is the fifth report to show a case of PAMM in pregnancy. Pecen et al. proposed that the development of PAMM in their case may have been influenced by a combination of anaemia, hypotension, and the hypercoagulable state associated with pregnancy [10]. In a similar vein, Chen et al. suggested that PAMM could be linked to autoregulatory changes in the retinal and choroidal vasculature, indicating that pregnancy itself may serve as a risk factor for the condition [11]. Coulon et al. found PAMM in pregnancy associated with hypercoagulability and elevated factor VIII levels. This could potentially lead to pregnancy complications including pre-eclampsia, intrauterine fetal death, severe intrauterine growth restriction, and placental abruption [3]. This case re-iterates the importance of looking for underlying hypercoagulopathies. In our case, the patient had a family history of thromboembolic events during pregnancy and was started on a low-molecular-weight heparin. Her blood work however was unremarkable. 

Our case is novel from others reporting PAMM in pregnancy because our patient suffered symptoms compatible with migraine with aura prior to her scotoma. Interestingly, this patient had no prior migraine history.

Migraine is a complex neurovascular disorder characterized by headaches that are often unilateral and throbbing with photosensitivity, sound sensitivity, and visual aura. The precise pathophysiology of migraine is still being explored, but it is thought to involve a complex interplay of neural, hormonal, and vascular factors. Migraines can rarely occur for the first time during pregnancy and this has been suggested because of hormonal imbalances, namely sharply increasing oestrogen levels which then later stabilize in the latter part of pregnancy [12]. Ophthalmic disorders associated with migraine include progression of normal tension glaucoma, retinal artery or vein occlusions, and ischaemic optic neuropathies [13]. Some of these events can occur during the acute migraine episode but also between attacks.

Three recent reports have shown PAMM developing after migraine [14-16]. Additionally, in a case series of nine PAMM patients by Chen et al., two of these patients had a history of migraines [17]. Finally, one case report showing PAMM in pregnancy also had a history of migraine although they had no reported recent attack before PAMM developed [18].

One theory for associating migraines with PAMM could be due to an underlying microangiopathy which makes them more susceptible to ischaemia. Studies have shown on OCT-A reduced vascular density in the superficial and deep capillary plexuses as well as enlarged foveal avascular zones in migraine sufferers [13]. This is an interesting finding; however, in our case, we felt it was quite peculiar that the patient had no prior history of migraines and it was her first episode that led to PAMM. Another proposed mechanism is that migraine attacks induce intermittent retinal arterial vasospasms, which can cause microvascular changes potentially near the fovea and predispose to ischaemia and cell destruction [16]. It will be interesting to see if further research can give us more answers on the underlying pathogeneses.

Overall, in our case, we believe there were combining risk factors at play for the development of PAMM. These included her migraine, pregnancy, a positive thromboembolic family history, and moderately high caffeine consumption.

## Conclusions

In conclusion, we report a case of PAMM in a young healthy patient after a migraine with an aura episode during her first pregnancy. Clinicians should be wary of persisting scotomas after migraines and work up with both SD-OCT and OCT-A to look for PAMM. Further, underlying systemic vascular risk factors should then be excluded.

Further research should be conducted to determine first whether there is a definite relationship between pregnancy, migraines, and PAMM and second to better understand the mechanisms that cause these events to occur.

## References

[1] Sarraf D, Rahimy E, Fawzi AA (2013). Paracentral acute middle maculopathy: A new variant of acute macular neuroretinopathy associated with retinal capillary ischemia. JAMA Ophthalmol.

[2] Nemiroff J, Kuehlewein L, Rahimy E (2016). Assessing deep retinal capillary ischemia in paracentral acute middle maculopathy by optical coherence tomography angiography. Am J Ophthalmol.

[3] Coulon SJ, Dedania VS (2023). Paracentral acute middle maculopathy associated with hypercoagulability in pregnancy. Retin Cases Brief Rep.

[4] Riazi-Esfahani H, Khalili Pour E, Fadakar K (2021). Multimodal imaging for paracentral acute maculopathy; the diagnostic role of en face OCT. Int J Retina Vitreous.

[5] Dansingani KK, Freund KB (2015). Paracentral acute middle maculopathy and acute macular neuroretinopathy: Related and distinct entities. Am J Ophthalmol.

[6] Nakamura M, Katagiri S, Hayashi T (2019). Longitudinal follow-up of two patients with isolated paracentral acute middle maculopathy. Int Med Case Rep J.

[7] Broyles H, Chacko J, Chancellor J, LoRusso F, Phillips PH, Mashayekhi A, Uwaydat S (2021). Paracentral acute middle maculopathy as the initial presentation of giant cell arteritis. J Neuroophthalmol.

[8] Virgo J, Mohamed M (2020). Paracentral acute middle maculopathy and acute macular neuroretinopathy following SARS-CoV-2 infection. Eye (Lond).

[9] Rahimy E, Kuehlewein L, Sadda SR, Sarraf D (2015). Paracentral acute middle maculopathy: What we knew then and what we know now. Retina.

[10] Pecen PE, Smith AG, Ehlers JP (2015). Optical coherence tomography angiography of acute macular neuroretinopathy and paracentral acute middle maculopathy. JAMA Ophthalmol.

[11] Chen X, Desai SJ, Baumal CR (2020). Paracentral acute middle maculopathy in pregnancy. Retin Cases Brief Rep.

[12] Pfaffenrath V, Rehm M (1998). Migraine in pregnancy: What are the safest treatment options?. Drug Saf.

[13] Güler Ö, Güler M, Tuğan Yıldız CB, Hakkoymaz H (2020). Are retinal and peripapillary blood flows affected during migraine attack?. Neuroophthalmology.

[14] Dasari VR, Selliyan A, Gratton SM (2022). Paracentral acute middle maculopathy in a patient with frequent migraine with aura: A case report. Retin Cases Brief Rep.

[15] Hall MN, Maleki A (2024). Paracentral acute middle maculopathy and cotton wool spots in a patient with ocular migraine: A case report. Eur J Ophthalmol.

[16] Monera Lucas CE, Escolano Serrano J, Romero Valero D, Castilla Martínez G, Pardo López S, Toledano Martos R (2022). Permanent damage of the inner retinal layers in a patient with migraine: A different case of paracentral acute middle maculopathy. Arch Soc Esp Oftalmol (Engl Ed).

[17] Chen X, Rahimy E, Sergott RC (2015). Spectrum of retinal vascular diseases associated with paracentral acute middle maculopathy. Am J Ophthalmol.

[18] Scott RA, Bhat N, Bindiganavile SH, Li HK, Lee AG (2022). Paracentral acute middle maculopathy in pregnancy. J Neuroophthalmol.

